# SAFE MED Quality Improvement Project: Supporting Availability and Functionality of Essential Monitoring Equipment for Drug Use in CAMHS (Child and Adolescent Mental Health Services) Outpatient Services in Oxfordshire

**DOI:** 10.1192/bjo.2025.10453

**Published:** 2025-06-20

**Authors:** Sureyya Melike Toparlak, Andreea Dumitrascu, Emma Fergusson, Marta Costa, Robert Chapman

**Affiliations:** Oxford Health NHS Foundation Trust, Oxford, United Kingdom

## Abstract

**Aims:** Main aim of this quality improvement project was to ensure that each outpatient CAMHS clinic room is equipped with the necessary, functional equipment for safe and comprehensive baseline and ongoing monitoring of patients prescribed antidepressant, antipsychotic, and ADHD medications, in line with NICE guidelines, the Maudsley Prescribing Guidelines and hospital guidelines.

**Methods:** The sites included in this project are Raglan house (single point of access), The Clock house (Community Service South Oxfordshire), Slade and Maple house (neurodevelopmental conditions outpatient service in Oxford).

Data collection was conducted with the help of a checklist to be used for each clinic room in outpatient CAMHS. All four sites were included in the data interpretation process each having 54 items in the checklist. It includes quantitative and qualitative data which are crucial to ensure standards and to meet requirements by the guidelines mentioned above. These items consist of three groups, physical health monitoring, infection prevention as well as privacy, confidentiality and comfort. Some of the items were window blinds and engaged/vacant for privacy and confidentiality; sanitiser and soap for infection prevention; stethoscope and height measurement tool for physical health.

**Results:** On average, 44% of checklist items were present on the sites, which means 56% items were not available. Of the present items, 96% were working well, whereas 4% were dysfunctional such as a clock with no battery, an unstable scale, a faulty thermometer and limited amount of sanitiser. Moreover, concerns were raised about shortage of rooms for routine and urgent appointments across multiple sites despite online and telephone appointments being offered. In addition, some of the rooms did not have appropriate lighting. The issues that pose immediate risk to patients’ safety were prioritised and reported to the estates. The rest is planned to be reported at the time this abstract was written.

**Conclusion:** Functional clinical equipment is essential to ensure patient safety. Efficient and active use of channels to report missing or dysfunctional items as well as regular maintenance and calibration of clinical items are key to excellent caring and safe care. All staff members are responsible to make sure that appropriate equipment is available.

